# From Diagnosis to Therapy: The Role of Virtual and Augmented Reality in Orthopaedic Trauma Surgery

**DOI:** 10.7759/cureus.29099

**Published:** 2022-09-13

**Authors:** Aditya Gupta, Ratnakar Ambade

**Affiliations:** 1 Orthopaedic Surgery, Jawaharlal Nehru Medical College, Datta Meghe Institute of Medical Sciences, Wardha, IND; 2 Orthopaedics, Jawaharlal Nehru Medical College, Datta Meghe Institute of Medical Sciences, Wardha, IND

**Keywords:** augmented reality, technology, rehabilitation, virtual reality, orthopaedic trauma surgery

## Abstract

By reducing procedure-related problems, advancements in computer-assisted surgery (CAS) and surgical training aim to boost operative precision and enhance patient safety. Orthopaedic training and practice have started to change as a result of the incorporation of reality technologies like virtual reality (VR), augmented reality (AR), and mixed reality (MR) into CAS. Today's trainees can engage in realistic and highly involved operational simulations without supervision. With the coronavirus disease 2019 (COVID-19) pandemic, there is a greater need for breakthrough technology adoption. VR is an interactive technology that enables personalised care and could support successful patient-centered rehabilitation. It is a valid and trustworthy evaluation method for determining joint range of motion, function, and balance in physical rehabilitation. It may make it possible to customise care, encourage patients, boost compliance, and track their advancement. AR supplementation in orthopaedic surgery has shown promising results in pre-clinical settings, with improvements in surgical accuracy and reproducibility, decreased operating times, and less radiation exposure. As little patient observation is needed, this may lessen the workload clinicians must bear. The ability to use it for home-based therapy is often available commercially as well.

The objectives of this review are to evaluate the technology available, comprehend the available evidence regarding the benefit, and take into account implementation problems in clinical practice. The use of this technology, its practical and moral ramifications, and how it will affect orthopaedic doctors and their patients are also covered. This review offers a current and thorough analysis of the reality technologies and their uses in orthopaedic surgery.

## Introduction and background

Over the past century, the world's health system has undergone a transformation thanks to the exponential expansion of processing power and imaging technologies, which have enhanced patient care from diagnosis to treatment. The most cutting-edge imaging and computing technologies have come together to create reality technologies, which have the potential to revolutionise many facets of the healthcare sector, particularly surgery. In contrast to virtual reality (VR), which immerses the user in a wholly artificial world, augmented reality (AR) adds information to real items in real-time [1]. Using the head-up display (HUD) to block the visual field is one of the alternate strategies. Oculus Rift (Facebook Technologies, LLC, Menlo Park, California, United States), which had achieved success in the commercial gaming industry, is an illustration of this. In the field of surgery, immersive virtual reality operating room training experiences have been tested.

Numerous experts contend that pilot training, which makes use of simulation before exposing students to real-world scenarios, is a good model for surgical training [2]. Some contend that before encountering clinical scenarios with patients, surgical trainees have "a behavioural imperative" to be exhibited to all those that may be "rationally effectively mimicked" [3]. Supporters contend that by using this strategy, instructors and students can concentrate on learning new information and skills instead of having to balance patients' safety [4]. The use of AR in interventional medicine has already been established in the fields of neuro- and visceral surgery. The implementation of AR in the field of orthopaedic surgery, which is the subject of this systematic review study, is receiving more and more attention. It is not surprising that there is a growing interest in AR in orthopaedics and trauma, given that orthopaedic surgical procedures routinely utilise visual data, that is images obtained one before and the other during surgery, and frequently involve instinctive steps, such as screw or graft installing, surgical procedures, and remediation of disfigurement, which can be visualised as firm relations in AR environments. As a result, the application of AR seems to be suited for such technical tasks [5]. These advancements in orthopaedic surgery have aimed to provide advantages to the surgical team while capturing improvements relevant to patient care. Technology is used in AR to add more information to the real world, such as the appearance of being in different locations [6].

## Review

Fundamental building blocks of VR and AR

VR exists as a method that saturates the viewer visually in a wholly unnatural, computer-generated environment. Mobile headsets are the most common type of standalone VR equipment. However, even in a simulated environment, the most cutting-edge VR experiences can allow for freedom of movement. The computer may potentially supply VR visual components in addition to fabricating false audio and other inputs. The VR experience can also be enhanced with specialised hand-operated controllers [7]. Contrary to VR technology, which immerses the user in an entirely artificial environment, AR technology superimposes a computer-generated picture over real-world surfaces to give the person a stereotypical representation. The optimum use of AR machinery would be via a head-mounted display (HMD) or on a mobile device, which would allow the surgeon to see the anatomical target without having to move to a different screen. Using specialised AR headsets like HoloLens (Microsoft Corporation, Redmond, Washington, United States) and Samsung HMD Odyssey+ (not available now; The Samsung Group, Suwon-si, South Korea), users are provided with digital content in real-time on a small screen that is placed right in front of their eyes [8].

The most recent development in reality-machine technology is mixed reality (MR). A stereographic picture is created in an MR system by fusing a real-world surface with a three-dimensional (3D) virtual replica created through diagnostic imaging scans such as computed tomography (CT) scans or magnetic resonance imaging (MRI) taken prior to surgery. Virtual objects aren't as clearly projected onto real-world surroundings when employing MR technology as they are with AR. However, the software helps the MR user to interact with both the real environment and the added digital material. The 3D picture (hologram) may be altered from the user's point of view by using webcams in collaboration with the viewfinder [9]. Orthopaedic and trauma surgery includes a variety of procedures, such as arthroscopy and the therapy of bone fractures. In fracture reductions, our main goal is to realign the broken bone fragments to their proper anatomical positions. Screws, plates, or implants may be necessary, depending on the type and complexity, to correct the ensuing lower composition [10]. When performing surgery in a true clinical environment, more practical independence has been demonstrated by the learner. Participants reported that the virtual presence of the teacher served as a safety net for the students, boosting their self-confidence in their own abilities [11].

The Implantation of Device or Implant

The 3D orientation of the orthopaedic surgeon is frequently used for placing instruments or implants. Two-dimensional (2D) information is provided by intraoperative fluoroscopy. In order to map the 2D radiographs to the 3D anatomy, the surgeon must do it mentally. By placing preoperative planning into the surgeon's field of vision or even by displaying the proper implant placement trajectories with overlays, AR systems reduce the reliance of the outcome on the surgeon's parameters. Based on preoperative CT data, Wu et al. projected the spinal bone structure at the back of a sufferer with approaching locations for vertebroplasty using a camera-projector AR system [12].

In a phantom investigation, Abe and colleagues simulated the insertion of needles into vertebral bodies [13]. The place and angles of insertion upon sufferers' preoperative CT images were identified. A webcam-equipped video see-through HMD (Moverio, Seiko Epson Corporation, Suwa, Nagano, Japan) was worn by the surgeon while performing the procedure. The webcam recorded everything it saw and sent it to a computer so it could be processed. The identification between the subject and the CT was performed using a few fluoroscopy pictures and many manual procedures [13]. Using the so-called camera-augmented mobile C-Arm system, which comprises a mobile C-Arm and a recording device attached near to the X-ray source, Navab et al. focused on AR-supported vertebroplasty. Because of the double mirror architecture used in this system's design, the optical and X-ray cameras' points of origin are almost identical. The main advantage of this technology was the ability to combine video recording frames out from the operative site with C-Arm fluoroscopy pictures without the need for image distortion [14]. Using an imaging system with AR capabilities, the CAMC method was further examined for the insertion of thoracolumbar pedicle screws [15]. Using an ultrasound-assisted registration technique, Ma et al. studied pedicle screw implantation. The patient's preoperative CT scan was registered using ultrasound, and an integral videography method was used to overlay surgical navigation [16].

Gains and advantages of VR and AR

Precision and Accuracy

This was attributable to enhanced, logical visual aids that made sure the operator placed the surgical tools precisely. As an illustration, consider a head-mounted gadget that displays a hologram of the precise location where a spinal needle should be inserted. Through an ocular see-through HMD, Gibby et al. superimposed the virtual path of needle implantation using computed CT data [11].

Efficiency in Terms of Time

The analyzer might have categorised it as proper because the needle spike was on the inside the joint capsule. The implementation of such technology thus seems to have the potential to considerably improve the accuracy and safety of orthopaedic surgery [17]. According to Alexander et al., percutaneous vertebroplasty required more time with standard fluoroscopy and AR-guided trocar implantation (642 seconds with AR vs. 336 seconds with standard fluoroscopy; range: 240e438; p 0.001 with AR). The difference between the first and last five time periods, however, was considerable, showing that as the surgeon became more accustomed to using augmented reality, there was a higher confidence in its use and less dependency on additional screening [18].

Decreased Unnecessary Radiations

The reduction of needless radiation exposure towards operating room employees was a frequent goal of development in AR technology. When compared to conventional intraoperative fluoroscopy, the use of AR to just provide 3D surface reformation via digitally recovered radiographs showed significant decrease in radiation exposure while enabling real-time visual display with no need for consistent fluoroscopy [19].

Pauly et al. employed a ceiling-mounted C-arm including an AR surgical navigation system to measure how much radioactive operating room staff in a hybrid operating room were exposed to. The staff members had 20 surgeries to have pedicle screws inserted while wearing an active personal radiation detector on their breast. These findings were compared to a benchmark radiation exposure level, or "worst case," as measured by a dosimeter mounted on a C-arm. The personnel-to-standard dosage ratio amounted to 0.05% for each procedure, and after several procedures, it further decreased to less than 0.01%, illustrating once more how technology advances with use [20].

Benefits in Education

The usage of AR/HUD as a teaching aid for orthopaedic surgery has a lot of potential to grow. The use of a HUD (Google Glass, Google LLC, Mountain View, California, United States) for perioperative instruction and interactions between a junior surgical team wearing the headset and a senior remote surgeon viewing the operator's surgical site via a computer or mobile device using the Google Hangouts app (Google LLC) was demonstrated by Armstrong et al. [21]. The students were able to assert more autonomous practical learning control while doing surgery in a genuine clinical setting. Participants said that the learner had greater confidence in their solo techniques because of the safety net provided by the professor's virtual presence.

Application and use in orthopaedic diagnosis

It is no longer possible to fully master all the abilities necessary to become fully competent as an orthopedist in a clinical setting alone. Due to rising resident training costs, declining resident working time, and ethical concerns about patient care, teaching trainees outside of the operating room is important [22]. VR can bridge these gaps in the present residency training by offering a substitute for further operational training. The system has the capacity to generate a highly interactive, realistic operational simulation with options for performance monitoring and analysis. Additionally, these simulators are easy to operate and don't need a trained professional to watch over and direct the pupil. Researchers found that residents who had access to VR completed surgeries more rapidly than those who did not in a trial using the minimally invasive surgical trainer in virtual reality (MIST-VR) laparoscopic simulator [23].

Sometimes, it becomes vital to visualise the inner workings of a joint to help with diagnosis or to support the surgeon during surgery. An answer to this issue is arthroscopy. By putting a tiny camera into the articular cavity of a synovial joint, it involves a minimally invasive investigation of interior structures like cartilage or ligaments. Results are shown on a screen outside the computer. A number of businesses created arthroscopy simulators that incorporate virtual reality and mannequins for training. The two most important ones at the moment in this field are ArthroS™ (VirtaMed, Zurich, Switzerland) and TraumaVision® (Swemac Innovation AB, Linköping, Sweden), which have been supported by numerous study publications [24]. In order to restore the proper anatomical position after some fractures, screws or other fixation must be inserted. One of the riskiest orthopaedic procedures is pelvic fracture repair, particularly in the elderly [25].

Usability of AR

Studies that visualised AR in orthopaedic surgery used head-mounted gadgets like Google Glass, but these methods had some drawbacks. First of all, after using the headset for an extended period of time, numerous consumers complained of visual discomfort. As the computer changed the deepest portion of the image, the consumer had to adapt one‘s eyes to a fixed focus distance of 2 m away, which led to vergence and visual fatigue- accommodation issues [26]. Even though the AR visuals are not explicitly superimposed on anatomical characteristics, several head-mounted technologies have the ability to draw the participant's attention away from the surgical site. As it might distract the surgeon, this would need to be addressed [27].

In darker settings, it can be difficult to see some AR graphics, that is holographic displays and overlaying systems, which might prove problematic in surgical units. To ensure that these real-world and AR pictures are readily visible or through head-mounted devices, more contrast and other colour designations should be established. Furthermore, issues like colour blindness need to be addressed [26,28]. Last but not least, the adoption of such technology would be encouraged by claimed or actual benefits to surgeons given the hard environment they work in. To improve patient outcomes and surgeon wellbeing, the burden of surgeons who routinely do high-risk and emergency surgeries must be reduced [29]. Because there hasn't been much progress beyond the use of radio-protective covers, it's critical to leverage technology to reduce operating times and fluoroscopy exposure [30].

The rehabilitation programme

The advantages of AR and VR may include an increase in the surgeon's focus on the operating room, a decrease in the risk of radiation exposure, through outpatient therapies including rehabilitation and a decrease in surgical field blockage [31,32]. A rehabilitation programme for orthopaedic trauma procedures entails different sets of repetitive exercises to help a broken bone regain mobility. The exercises must be done steadily and consistently to be successful. To monitor patient activity and the calibre of the workouts performed, a good system should provide adequate feedback [33]. The reasons for stopping the rehabilitation process include discomfort, boredom, stagnation, or phoney recovery symptoms. As VR systems advance, patients may now complete these tasks more easily, giving the therapy a joyful element [34]. Patients can do exercises at home with the assistance of an expert if necessary, making telerehabilitation a popular field for the application of VR and AR environments. In our opinion, the reader should concentrate on adopting VR for motor rehabilitation by incorporating methods from various medical specialities [35].

Future aspects of AR

Despite the popularity of AR for surgical navigation, there is still some debate regarding the intraoperative usage of HMDs and their place in clinical care. Although few clinical research and pilot studies have shown its usefulness in bench and cadaver models, surgeons still face a number of logistical and moral dilemmas when integrating this technology into patient treatment. HMDs could soon be utilised to display essential information, photographs, and even fluoroscopy images in the surgeon's field of view if bigger clinical studies indicate improvements over current practice. More sophisticated tracking software needs to be developed in order to maximise the usefulness of these devices in the operating room. While optical tracking is an accurate method of measuring position during surgery, it does have the drawback of requiring [36].

## Conclusions

Reality technologies hold considerable promise as an improvement over conventional computer-assisted surgical techniques for orthopaedic training and practice. The ability to visualise patient data in real-time, better preoperative planning, and increase the accuracy and precision of interventions lead to an improvement in the calibre of care and patient outcomes. Reality technologies offer a fresh approach to live evaluation and remote coaching from the standpoint of education. On the other hand, AR is more equipped to help surgeons. Although they both have great potential, they haven't been thoroughly studied to entirely replace current practises. For these platforms to be finally implemented in a real-time surgical scenario, new, more potent, and accurate instruments must be developed.

Additionally, since organ features may be approximated and genuine surgical instruments can be used, AR with 3D projections may be a better training tool than VR simulators. Due to this feature, this technology is currently popular in medicine. In fact, it is feasible that the invention and application of breakthrough technologies will quicken given the rapidity of change seen during the coronavirus disease 2019 (COVID-19) pandemic. Even so, it will be crucial to make sure that any such acceptance of innovation is grounded in safety and better results, and that the rate of change is moderated with the goal of achieving those results.
